# Pleiotrophin Gene Therapy for Peripheral Ischemia: Evaluation of Full-Length and Truncated Gene Variants

**DOI:** 10.1371/journal.pone.0061413

**Published:** 2013-04-22

**Authors:** Qizhi Fang, Pamela Y. Mok, Anila E. Thomas, Daniel J. Haddad, Shereen A. Saini, Brian T. Clifford, Neel K. Kapasi, Olivia M. Danforth, Minako Usui, Weisheng Ye, Emmy Luu, Rikki Sharma, Maya J. Bartel, Jeremy A. Pathmanabhan, Andrew A. S. Ang, Richard E. Sievers, Randall J. Lee, Matthew L. Springer

**Affiliations:** 1 Cardiovascular Research Institute, University of California San Francisco, San Francisco, California, United States of America; 2 Division of Cardiology, University of California San Francisco, San Francisco, California, United States of America; 3 Eli and Edythe Broad Center of Regeneration Medicine and Stem Cell Research, University of California San Francisco, San Francisco, California, United States of America; Institute of Clinical Medicine, National Cheng Kung University, Taiwan

## Abstract

Pleiotrophin (PTN) is a growth factor with both pro-angiogenic and limited pro-tumorigenic activity. We evaluated the potential for PTN to be used for safe angiogenic gene therapy using the full length gene and a truncated gene variant lacking the domain implicated in tumorigenesis. Mouse myoblasts were transduced to express full length or truncated PTN (PTN or T-PTN), along with a LacZ reporter gene, and injected into mouse limb muscle and myocardium. In cultured myoblasts, PTN was expressed and secreted via the Golgi apparatus, but T-PTN was not properly secreted. Nonetheless, no evidence of uncontrolled growth was observed in cells expressing either form of PTN. PTN gene delivery to myocardium, and non-ischemic skeletal muscle, did not result in a detectable change in vascularity or function. In ischemic hindlimb at 14 days post-implantation, intramuscular injection with PTN-expressing myoblasts led to a significant increase in skin perfusion and muscle arteriole density. We conclude that (1) delivery of the full length PTN gene to muscle can be accomplished without tumorigenesis, (2) the truncated PTN gene may be difficult to use in a gene therapy context due to inefficient secretion, (3) PTN gene delivery leads to functional benefit in the mouse acute ischemic hindlimb model.

## Introduction

Therapeutic vascular growth (i.e., angiogenesis and arteriogenesis) induced by genes or proteins has been suggested as a potential approach to improve blood flow by the induction of neovascularization to ischemic tissue [1]. However, the relative merits of the angiogenic factors currently in use continue to be the subject of much debate. In addition, vascular endothelial growth factor (VEGF), the most common angiogenic factor in clinical tests, can lead to undesirable consequences such as hemangiomas and atherosclerotic lesions if expressed at too high a level in animal models [2], [3], [4], [5], [6], [7] even if VEGF concentrations become too high in extremely localized regions on a microscopic level [8], [9]. Members of the FGF family have shown intriguing possibilities [10], [11], but therapeutic angiogenesis has yet to become a clearly beneficial clinical tool. Consequently, as the first generation of angiogenic factors continues to be evaluated in the clinic, and novel strategies for delivery of factor combinations are developed [12], [13], [14], it remains important to consider other angiogenic factors as they are discovered and to evaluate their therapeutic potential.

Pleiotrophin (PTN) is a cytokine that plays multiple roles involved in neurite outgrowth and angiogenic response to ischemic injury in the brain and heart, mediated by at least two receptors on endothelial cells [15], [16]. It is also expressed in a variety of tumors. PTN endogenous expression and exogenous exposure is reported to drive monocytes toward vascular endothelial phenotypes [17], [18] and we have demonstrated that PTN is a chemoattractant for circulating angiogenic cells (CACs, alternatively called endothelial progenitor cells), in a similar fashion to chemoattractants VEGF and SDF-1α [19]. Because of these characteristics, PTN may present an attractive tool for angiogenic gene therapy.

**Figure 1:**
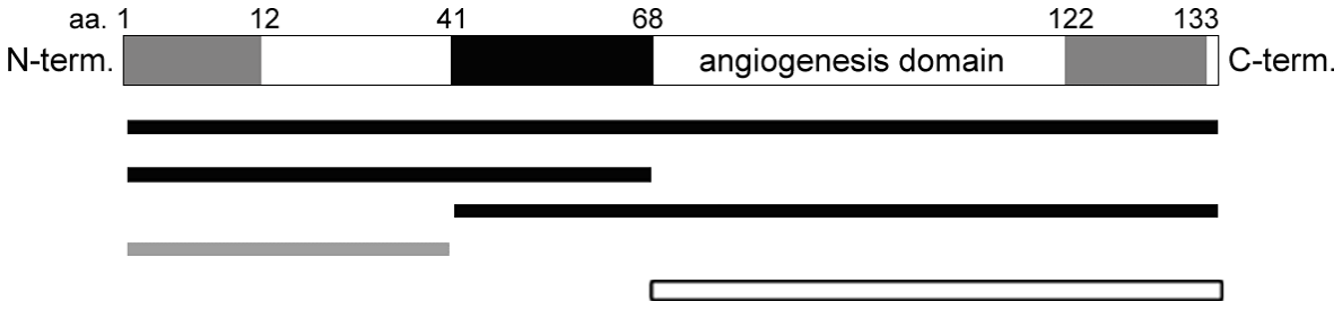
PTN angiogenesis domain and transformation domain. Full length PTN or any mutant that contains the black domain and either grey domain (represented by black lines) can transform cells and increase vascularity of tumors. Mutants that contain the N-terminal grey domain but no black domain (represented by grey line) are nonfunctional. A mutant that contains the C-terminal domain and no black domain (white line with black outline) does not transform cells, but does increase vascularity of tumors.

PTN has a potential downside, however, in that it can also exhibit transforming ability when over-expressed in cultured cells. This is cause for concern in any cell-mediated gene delivery approach. PTN possesses distinct domains that induce angiogenesis and transform cells, and a truncated mutant of PTN containing only the “angiogenesis domain” has been shown to increase angiogenesis in pre-existing tumors without having intrinsic transforming ability [15], [20] (**Figure 1**). Such a truncation mutant may present a safer alternative to the full-length PTN for therapy.

We tested whether the full-length and truncated PTN (“PTN” and “T-PTN”) gene variants could be used to induce angiogenesis or functional benefit in mouse skeletal muscle and myocardium through localized myoblast-mediated gene delivery, and evaluated the safety profile of PTN gene delivery on multiple levels. Parallel research was carried out in both cardiac and skeletal muscle because common mechanisms may manifest themselves differently in these two tissue environments. Similarly, gene delivery was attempted in these tissues under both normal and ischemic/post-MI conditions because the respective different tissue environments may respond differently to the PTN expression. We show here that T-PTN exhibits aberrant processing that prevents efficient secretion, but that full-length PTN gene delivery via primary myoblasts is safe and leads to functional benefit in a mouse model of hindlimb ischemia.

## Results

### Truncated PTN accumulates in the endoplasmic reticulum

**Figure 2:**
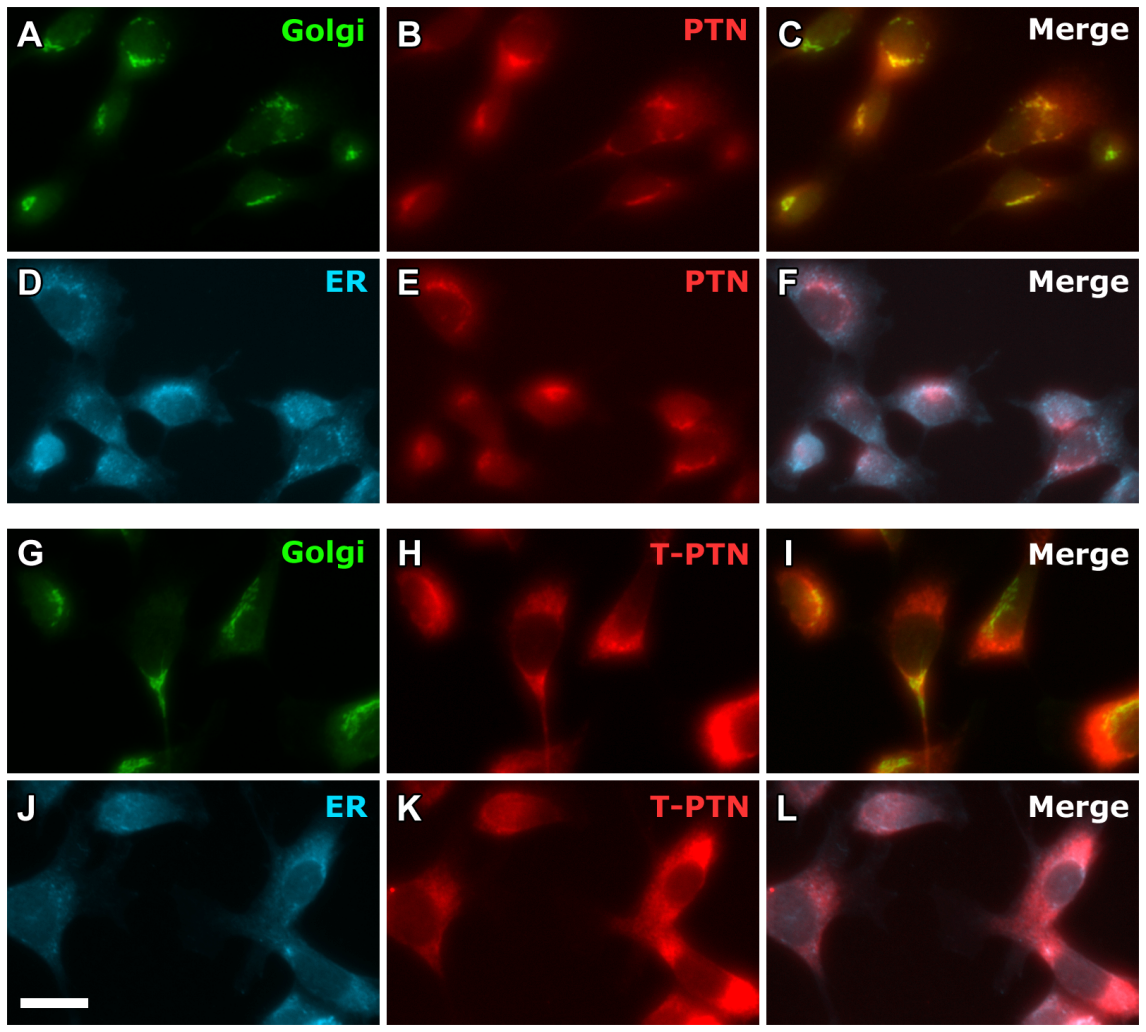
Truncated PTN accumulated in the endoplasmic reticulum. A,G) Golgi staining, D,J) ER staining, B,E,H,K) PTN staining, C,F,I,L) merged images, showing colocalization of PTN with the Golgi apparatus and of T-PTN with the ER. Scale bar = 20 µm.

**Figure 3:**
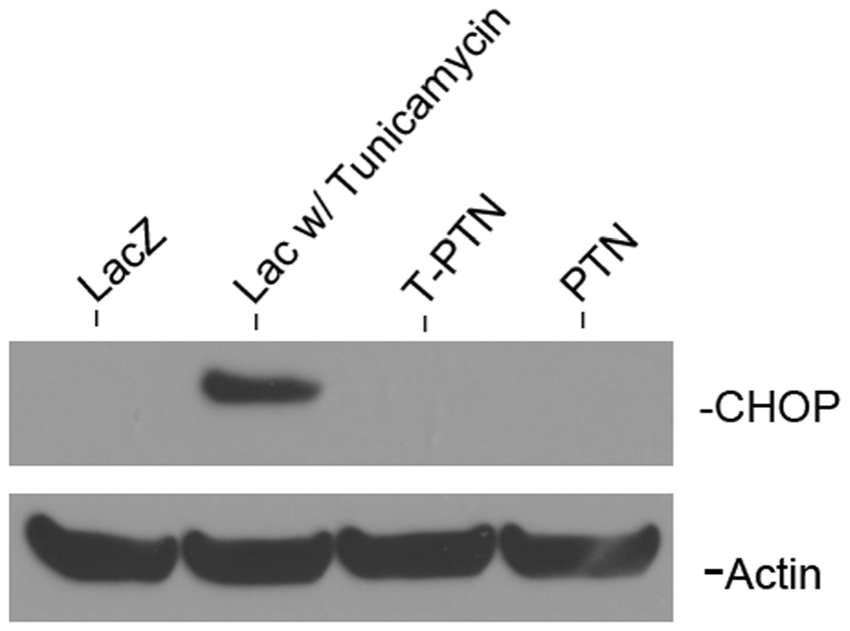
Myoblasts expressing truncated PTN showed no sign of ER stress. ER stress-induced CHOP expression was detected in LacZ myoblasts treated with 5 µg/ml tunicamycin as a positive control, while no expression was found in untreated T-PTN, PTN, and LacZ myoblasts.

Based on the published sequence of human PTN, which has not been observed to elicit an immune response in rats while still inducing angiogenesis, we generated retrovirus containing the full-length and truncated *Ptn* coding sequences. Upon retroviral transduction of PTN or T-PTN constructs into myoblasts already expressing LacZ, immunostaining for PTN in cultured cells showed similar brightness, implying that similar amounts were being made of each. However, when PTN secreted into culture media was measured from 8 clonal isolates of each population, the range of concentrations from T-PTN isolates was ∼10-fold less than those from PTN isolates (Table 1). Co-staining for Golgi and ER markers revealed that while full-length PTN was localized in the Golgi, implying normal secretion, T-PTN had a different staining pattern that colocalized with ER, implying that the truncated form is improperly folded or processed and gets trapped in the ER (**Figure 2**). However, as shown in **Figure 3**, we did not observe evidence that this secretion defect led to ER stress, based on lack of expression in T-PTN myoblasts of of the ER stress marker CCAAT-enhancer-binding protein homologous protein (CHOP) [21], [22]. Furthermore, there was no observable defect in growth rate of the T-PTN myoblasts relative to those expressing full-length PTN (not shown).

**Table 1:** PTN concentration secreted by different myoblast clones.

PTN Concentration (ng/day/10^6^ cells)	PTN	T-PTN
Clone 1	6.41	0.46
Clone 2	6.82	0.54
Clone 3	9.31	1.99
Clone 4	4.63	1.83
Clone 5	5.78	1.49
Clone 6	18.17	2.24
Clone 7	7.80	0.32
Clone 8	15.50	0.55
Mean±SD	9.30±4.58	1.17±0.74

The range of concentrations from T-PTN isolates was roughly 10-fold less than those from PTN isolates.

### No evidence of tumorigenesis from full-length PTN expression in myoblasts

**Figure 4:**
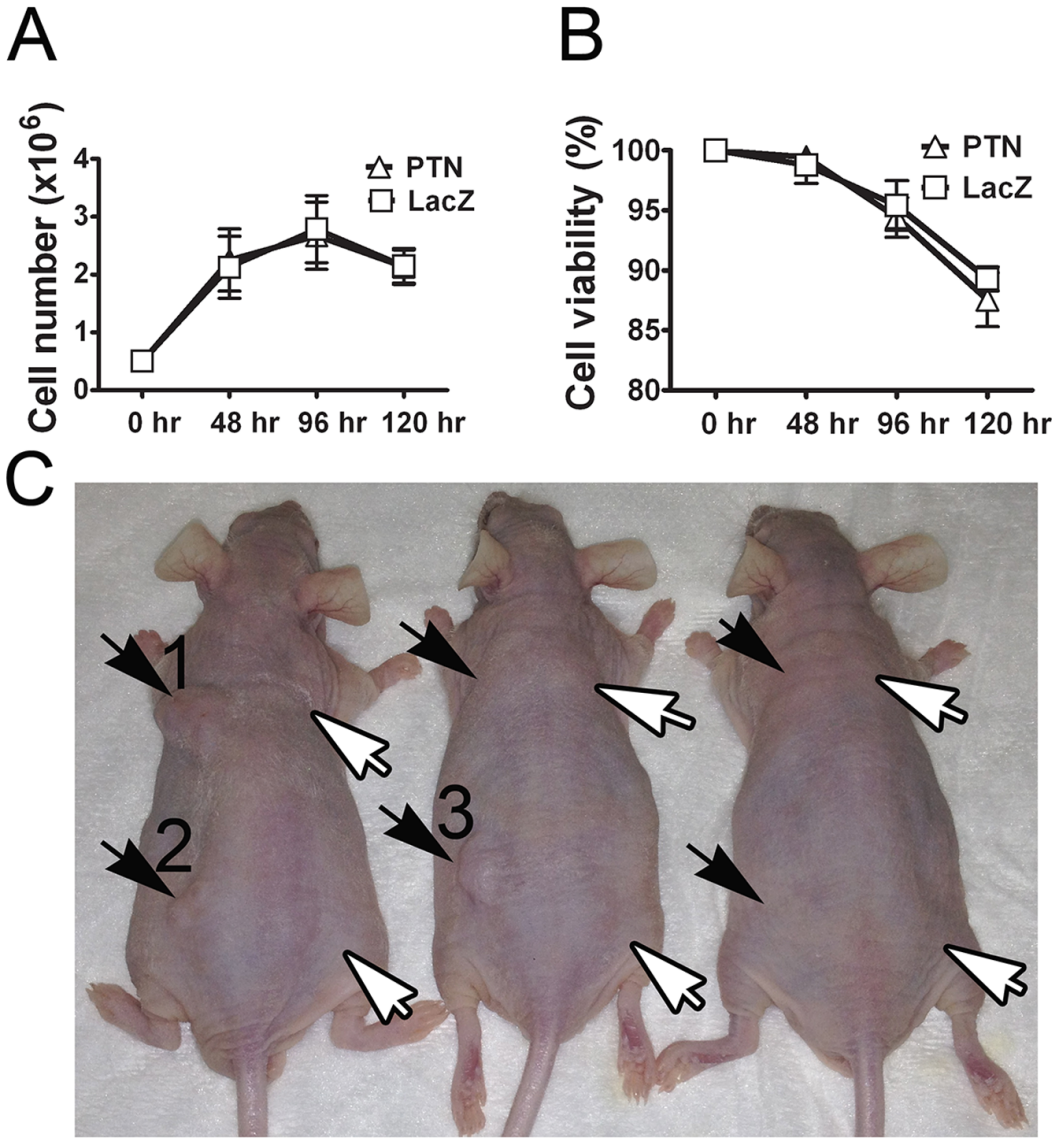
No evidence of tumorigenesis from full-length PTN expression in myoblasts. A) When PTN myoblasts and lacZ myoblasts were plated at equal densities and grown past confluence, the cells proliferated and declined at equal rates regardless of PTN expression. B) There was no difference between the cell types in percent viability after contact inhibition. C) The positive control MDA-MB-231 breast cancer cell line induced subcutaneous tumors at 3/6 implantation sites after 4 weeks (black arrows), whereas implantation of PTN myoblasts resulted in no detectable tumor formation (0/6; white arrows) in this experiment, and 0/5 after 10 weeks in a separate experiment (not shown). 1×10^5^ cells were injected in 10 µl PBS for anterior injections and 10 µl Matrigel for posterior injections. Animals are shown 4 weeks post injection. Tumor #1 = 1×0.8×0.5 cm; tumor #2 = 0.3×0.3×0.2 cm; tumor #3 = 0.7×0.9×0.5 cm.

Myoblasts expressing PTN and LacZ (PTN myoblasts) or LacZ alone (LacZ myoblasts) were assessed for tumorigenic potential by two different assays. (1) When PTN or LacZ myoblasts were seeded at equal density and grown beyond confluence after 48 hr post-plating, no significant differences were observed in final cell number between the types of myoblasts (**Figure 4A,B**). Both PTN and lacZ myoblasts expanded from 0.5×10^6^ to 2.6±0.4×10^6^ and 2.7±0.6×10^6^, respectively, in 96 hr, demonstrating that PTN did not bestow a growth advantage. Furthermore, the decrease of cell viability after confluence was also similar in the two cell types, demonstrating that PTN did not bestow a survival advantage past the point of contact inhibition. (2) PTN myoblasts and positive control MDA-MB-231 breast cancer cells were implanted subcutaneously into nude mice (n = 6 injection sites/cell type). Subcutaneous tumors were detected 4 weeks after injection of the MDA-MB-231 cells, whereas no tumors were detected after injection of PTN myoblasts (**Figure 4C**). Another group of nude mice was similarly injected with PTN myoblasts and LacZ myoblasts for long-term follow up, leading to no detectable tumors after 10 weeks (n = 5 injections per cell type). Moreover, as described below, tumors were not observed in muscle into which PTN myoblasts were implanted. Thus, the extra tumorigenic potential by PTN expression reported in some other systems does not appear to be present when expressed by myoblasts. Subsequent *in vivo* experiments were performed only with myoblasts expressing full-length PTN.

### Implantation of PTN-expressing myoblasts leads to vascular changes and functional benefit in ischemic skeletal muscle

**Figure 5:**
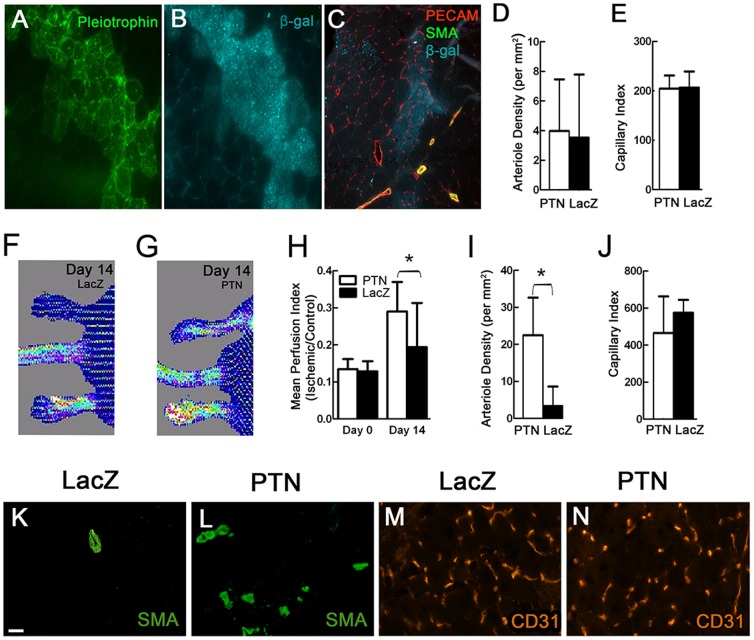
PTN-expressing myoblast implantation in normal and ischemic leg muscle. A,B) PTN staining and β-gal staining of a single section from non-ischemic mouse leg muscle implanted with myoblasts expressing both PTN and LacZ genes confirms that PTN is expressed in muscle fibers after myoblast fusion. C) The merged image shows triple staining for capillaries, smooth muscle cells (SMA = smooth muscle actin), and β-gal of a similar section. While some visible increases in vascularity occurred at some implantation sites relative to the surrounding tissue as pictured here, this happened in control LacZ implantation sites as well. Bar = 50 µm. D,E) No significant differences in arteriole density and capillary index were observed in non-ischemic muscles injected with PTN myoblasts versus LacZ myoblasts (n = 5). F,G) Representative laser Doppler images at 14 days showing a significant increment in skin perfusion at the feet after PTN myoblast treatment, in comparison with the control LacZ myoblast group (n = 12 for PTN, n = 10 for LacZ). H) Quantification of skin perfusion at feet of ischemic limbs based on laser Doppler measurements. *p<0.05. I) A significantly higher number of arterioles were detected in ischemic leg muscle implanted with PTN myoblasts than with LacZ myoblasts (n = 4), *p<0.05. J) No significant difference was observed in capillary index (n = 4). Smooth muscle actin (SMA) staining of arterioles (K,L) and CD31 staining of capillaries (M,N) in representative sections from ischemic leg muscle implanted with PTN myoblasts *vs*. LacZ myoblasts. Scale bar = 25 µm.

To assess the effects of PTN gene delivery to hindlimb skeletal muscle, we injected the PTN and LacZ myoblasts into non-ischemic SCID mouse leg muscle (n = 5 legs per group) to enable the myoblasts to fuse into the pre-existing muscle fibers, leading the fibers to express the recombinant proteins [2], [23], [24], [25]. SCID mice were used to prevent a slight chronic rejection of bacterial β-galactosidase protein that can occur in immunocompetent mice. The muscle fibers into which the myoblasts fused stained positive for both PTN and β-galactosidase (**Figure 5A–C**). Immunostaining for capillaries and arterioles (**Figure 5D,E**) revealed no significant difference in vascularity in the region of non-ischemic muscle injected with PTN cells *vs.* control cells (capillary vessel area index of 204.3±23.8 *vs*. 206.7±28.9; p = 0.90 and arteriole density of 4±3.1 *vs*.3.5±3.8 per mm^2^; p = 0.84).

A similar treatment was applied in a mouse hindlimb ischemia model based on unilateral ligation of the femoral artery, with myoblasts implanted in the vicinity of the ligation as has proven effective for VEGF-expressing myoblasts [9]. As detailed in **Figure 5F–H**, at 14 days post-surgery and cell implantation, ischemic limbs injected with control LacZ myoblasts showed markedly reduced perfusion relative to the non-ischemic contralateral leg (19.4±11.9%, p<0.01), confirming that the surgical procedure provided sustained blood flow reduction, and reflecting the expected endogenous angiogenic response relative to Day 0. Ischemic limbs implanted with PTN-expressing myoblasts showed a greater increase in perfusion over 14 days that was less reduced relative to the non-ischemic contralateral leg (29.0±8.0%, p<0.05), reflecting greater recovery during that time. While the laser Doppler measurements revealed significantly higher skin perfusion at feet of ischemic limbs injected with PTN myoblasts than those injected with LacZ myoblasts (**Figure 5H**), improvement in relative regional blood flow determined by 15 µm fluorescent microspheres counted in ischemic muscle normalized to contralateral non-ischemic muscle did not reach significance (0.63±0.60 (n = 10) *vs*. 0.65±0.20 (n = 11); p = 0.93). However, the quantification of vascular density by immunofluorescence staining revealed that PTN-expressing myoblasts significantly increased arteriole density (22.4±10.2 *vs.* 3.3±5.3 per mm^2^; p<0.05), but not capillary area density index (464.8±197.5 *vs.* 564.2±81.4; p = 0.34), at the implantation site compare to LacZ alone (**Figure 5I–N**).

### Implantation of PTN-expressing myoblasts into myocardium does not influence vascularity

**Figure 6:**
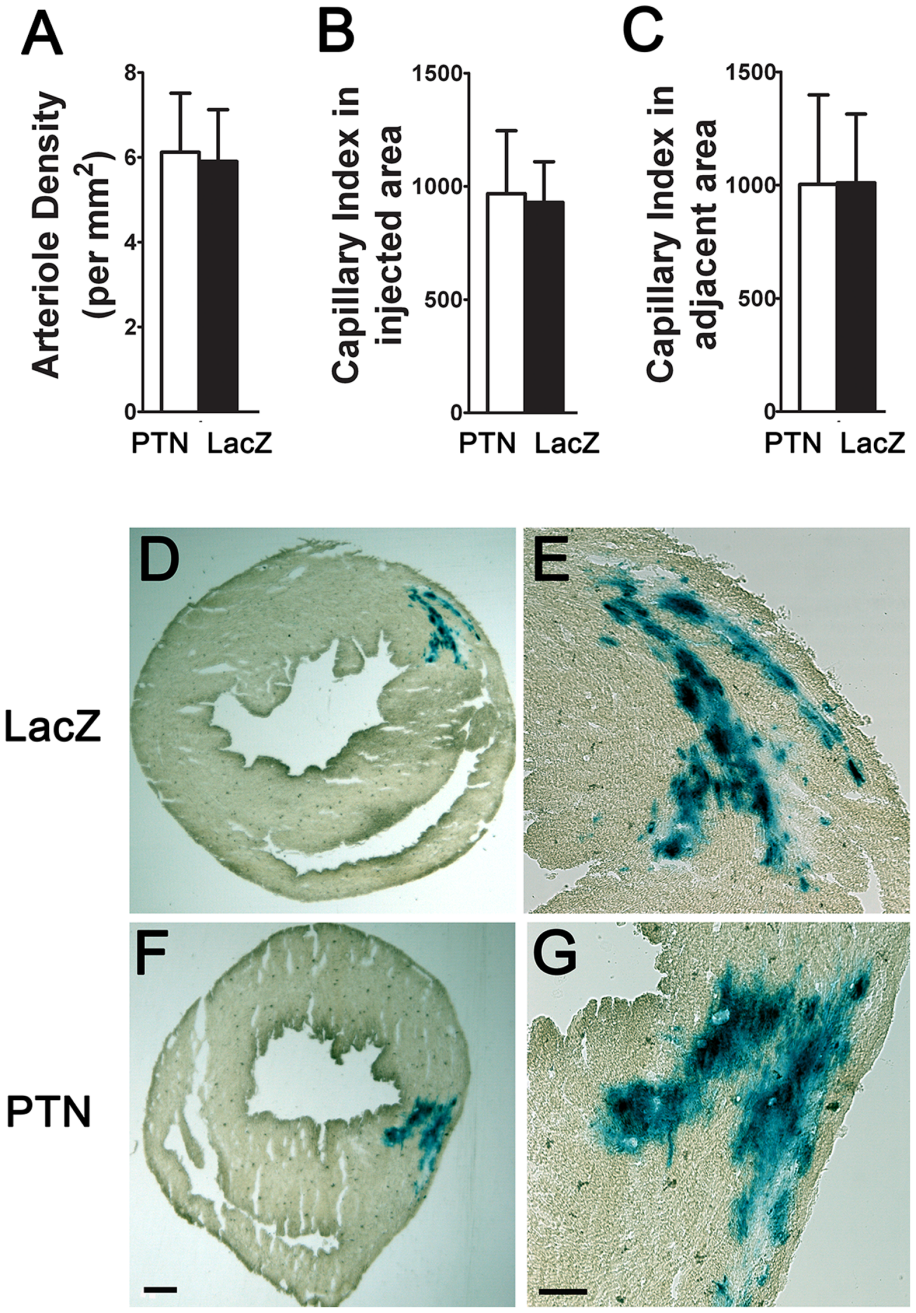
PTN myoblast implantation in myocardium. A) Arteriole density, B) capillary index in the injected area, and C) capillary index in the adjacent area exhibited no differences in mice implanted with PTN myoblasts *vs.* LacZ myoblasts (n = 5). X-gal staining confirmed the successful implantation of LacZ myoblasts (D) and PTN myoblasts (F). B,G) Higher magnification pictures of the implantation sites in (F) and (B). Scale bar in F = 0.5 mm, scale bar in G = 0.2 mm.

To assess the effects of PTN gene delivery to the heart, we implanted myoblasts expressing PTN and LacZ, or LacZ alone, into healthy myocardium. Histological evaluation at 14 and 28 days post-implantation detected no differences in capillary morphology, capillary area density or length density indices, or quantity of arterioles in either setting (**Figure 6**).

### Smooth muscle cells migrate toward PTN and endothelial cells

**Figure 7:**
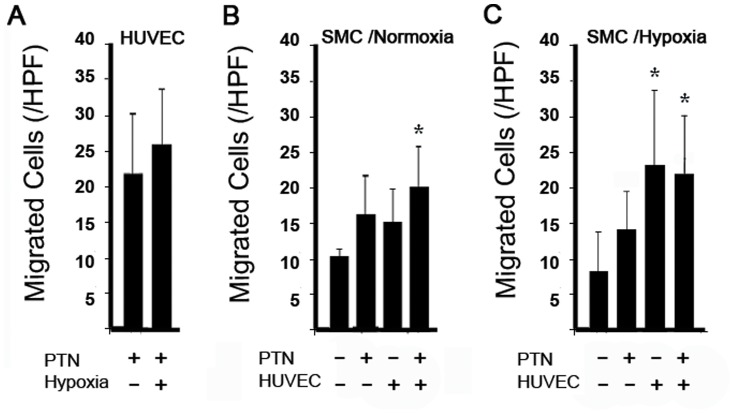
HUVECs and SMCs Migration assay. A) Hypoxia did not significantly increase migration of HUVECs toward to PTN. B) Under normoxic conditions, SMCs migrated toward HUVECs plus PTN. C) Under hypoxic conditions, SMCs migrated toward HUVECs alone and toward HUVECs plus PTN. *p<0.05.

In order to gain insight into PTN's apparent requirement for ischemia and preferential induction of new arteriole formation specifically, we examined whether hypoxic conditions bestowed pro-arteriogenic effects on cultured human umbilical vein endothelial cells (HUVECs) or human saphenous vein smooth muscle cells (SMCs). We previously showed that, under normoxic conditions, HUVECs migrate toward PTN in culture while SMCs do not. Thus, we performed similar experiments, under both normoxic and hypoxic conditions, which revealed the following. The response of HUVECs to PTN did not differ significantly under normoxic vs. hypoxic conditions (**Figure 7A**). Under normoxic conditions, SMCs did not migrate significantly toward PTN alone or to HUVECs alone, but migration to the combination of PTN and HUVECs achieved significance, suggesting that PTN's effects on HUVECs may increase attraction of SMCs (**Figure 7B**). Interestingly, under hypoxic conditions, SMCs migrated to HUVECs even in the absence of PTN (**Figure 7C**; see next section for discussion of this curious finding).

## Discussion

We undertook this study with the expectation that cell-based PTN gene delivery might entail a potential transformation risk. Expression of the truncated gene that lacks the tumorigenesis domain has been reported to be pro-angiogenic: therefore T-PTN and full PTN were studied in parallel. Full PTN was expressed in a normal Golgi staining pattern and secreted into the culture medium. T-PTN was expressed and stained in the cells as brightly as the full PTN, but with a different more diffuse staining pattern and far less was secreted into the medium. Co-localization with markers for Golgi and ER suggests that T-PTN misfolded and was retained in the ER. Notably, the truncation of the tumorigenesis domain was engineered in such a way that the leader peptide and signal sequence were intact, so the protein would be expected to undergo correct targeting and translocation into the lumen of the rough ER. However, the complete protein appears to have become stuck at that stage, either by misfolding in the lumen such that continued trafficking to the Golgi apparatus was impaired, or perhaps by a subsequent block in complete translocation into the ER lumen. Therefore, this truncation mutant of PTN is unlikely to be effective for therapeutic gene delivery, either by gene expression in implanted cells or by direct gene delivery to host tissue.

However, unlike results reported for tumor cells, we were unable to detect tumorigenic potential by myoblasts expressing PTN by implantation into nude mice or by assessment of growth/survival in culture. Notably, we have not observed tumors histologically in muscle implanted with PTN myoblasts. It is possible that myoblasts and skeletal muscle are simply less disposed to transformation by PTN than the NIH 3T3 cells that were shown to be transformed by Zhang *et al.* [26]. Thus, the inability to use the truncated PTN mutant gene to avoid transformation is counterbalanced by the apparent lack of transformation in the muscle. These results imply that PTN gene delivery does not need to rely on use of mutant PTN sequences lacking the transformation domain, but instead can be attempted using the native, full-length form. Clearly, these results will need to be confirmed for any different PTN gene delivery system envisioned for potential therapeutic use, but the current study benefits from the extra *ex vivo* step that enables transduced cells to be evaluated for effects on growth properties under pro-growth conditions before the genetic modification is introduced into differentiated tissue.

We have reported here that PTN-expressing myoblasts improved perfusion in a hindlimb ischemia model. Other groups [17], [18] have shown that monocytes that express or are exposed to PTN exhibit endothelial characteristics after injection into mice and can be therapeutic in ischemic hindlimbs, presumably via a mechanism related to endothelial behavior. In our case, the myoblasts expressing PTN differentiate into muscle (as confirmed in **Figure 5A–C**); thus, the beneficial effects of PTN after myoblast-mediated gene delivery are presumably due to production and secretion of the protein to the surrounding tissue. Despite difficulties in large-scale genetic alteration of muscle by myoblast transplantation, this result holds potential clinical relevance in that the use of muscle-derived stem cells is reported to result in a more robust and wide-spread dissemination through tissue [27], [28]; thus, a cell-mediated delivery of the PTN gene for treatment of peripheral artery disease remains an intriguing possibility.

While the implantation of PTN-expressing myoblasts had no detectable effect in non-ischemic hindlimb skeletal muscle relative to control myoblasts, implantation of PTN myoblasts in the quadriceps of ischemic legs led to an increase in local arteriole density with no increase in local capillary density, and an improvement in laser Doppler perfusion score in the foot despite no increase in microspheres trapped in the local capillary bed. These results are consistent with the interpretation that PTN in this system stimulated thigh arteriogenesis without inducing thigh angiogenesis, thus not leading to any increase in capillaries nor perfusion in the thigh as measured by microspheres, but the increase in thigh arterioles resulted in greater blood flow to the lower leg and increased skin perfusion at the foot detectable by laser Doppler. The effect on arterioles without effect on capillaries is reminiscent of what we have observed upon PTN plasmid injection into rat myocardium, which has consistently led to increases in arterioles while capillary changes are only sometimes observed [29]. It is important to note that “angiogenic” gene therapy frequently induces arteriole formation, with or without changes in capillary density [30], [31].

The observation that PTN-expressing myoblasts induced functional benefit in ischemic muscle but not non-ischemic muscle stands in contrast to the effects of similarly delivered VEGF-expressing myoblasts, which we have shown to induce robust endothelial growth in normal and ischemic muscle alike [2], [8], [9], [30], [32]. Ischemic muscle is more receptive to angiogenic and arteriogenic signaling in general, in that not only angiogenic factors but also their receptors are upregulated under ischemic conditions [33], [34]. In the case of PTN, at least one receptor (RTPT-β) is activated under hypoxic conditions by hypoxia-inducible factor-2 [35], [36]. We previously reported that PTN attracts HUVECs but not SMCs [19]. In the current study, hypoxia did not increase the attraction of HUVECs. Under hypoxic conditions, we observed attraction of SMCs to HUVECs with or without PTN, while attraction of SMCs to HUVECs under normoxic conditions reached signficance only in the presence of PTN. While seemingly paradoxical given that PTN increased arteriole formation only under hypoxic conditions, it is possible that PTN's attraction of endothelial cells, and the apparent facilitation of SMC attraction to HUVECs under hypoxic conditions, combine to surpass a threshold of some manner to induce extra arterioles in PTN-exposed ischemic muscle. Clearly, ischemia in vivo is a more complex condition than hypoxia in vitro, this interesting set of findings will be the subject of future investigations.

Similarly, we observed no effect on the vasculature from implantation of PTN-expressing myoblasts into non-ischemic heart, again in contrast to the effects of VEGF-expressing myoblasts in non-ischemic heart, which we have shown to be dramatic [3]. We did endeavor to implant PTN-expressing myoblasts into myocardium post-myocardial infarction (MI) with no effects detectable either by histology or by left ventricular functional analysis, despite our having considerable experience with intramyocardial cell implantation in mice [37], [38], [39], [40]. However, we have opted to not include these results because in contrast to our hindlimb ischemia model, the surgical induction of MI by ligation of the coronary artery is not a true ischemia model. Rather, it creates temporary ischemia in a region that then quickly dies, and myoblasts are implanted into the infarct border zone and spread to remote myocardium. Thus, a true evaluation of the effects of PTN-expressing myoblast implantation into ischemic myocardium will require a larger animal model or a gradual coronary artery occlusion rodent model.

In conclusion, we have demonstrated that PTN gene delivery via intramuscular implantation of genetically engineered myoblasts can be accomplished without tumorigenesis in mice, that use of a truncated PTN mutant lacking the transformation domain is neither effective nor necessary, and that myoblast-mediated PTN gene delivery can improve perfusion and tissue salvage in ischemic hindlimb muscle.

## Materials and Methods

### Construction of MFG-PTN retrovirus and genetic engineering of primary mouse myoblasts

The human PTN cDNA clone was synthesized *de novo* commercially (Blue Heron Biotechnology, Bothell, WA). PCR was used to engineer BspHI and BamHI sites at the 5′ and 3′ ends, respectively. The BspHI–BamHI fragment was excised and ligated into the standard NcoI (compatible with BspHI) and BamHI sites in an MFG retroviral plasmid to create pMFG-PTN. Quickchange (Stratagene, La Jolla, CA) loop-out was used to create the truncation mutant within pMFG-PTN, removing 204 bases from amino acid position 33 through 100. The ligation product was sequenced across the junctions and through the open reading frame to confirm the absence of PCR-related mutations. The resulting plasmid, pMFG-T-PTN, and pMFG-PTN were individually transiently transfected into Phoenix packing cells (supplied by G. Nolan) [41], [42] and retroviral supernatants were frozen on dry ice. Primary myoblasts already expressing *LacZ* from a retroviral promoter [43] were transduced at high efficiency [44] with four successive exposures to MFG-PTN or MFG-T-PTN virus. These processes resulted in parental PTN and T-PTN myoblasts that were 60–70% PTN-positive cells determined by immunofluorescence. Single cells from the PTN and T-PTN parental populations, which vary with respect to recombinant gene expression, were isolated by flow cytometry, and expanded into monoclonal populations. To quantify PTN secretion, medium was harvested from PTN and T-PTN cells after 6 hr of incubation and was then filtered and stored at −80°C, and the cells were counted. Thawed culture supernatants were analyzed in triplicate using a non-commercial ELISA in which plates were coated with anti-human PTN capture antibody (AF-252-PB, 2 ug/ml, R&D Systems, Minneapolis, MN) overnight, samples were incubated in the wells for 90 min, and bound PTN was detected by incubation with a biotin conjugate of the same antibody (BAF-252, 0.2 ug/ml, R&D Systems) for 60 min. Detection was amplified by incubation with Streptavidin-HRP, and visualized with a commercial HRP substrate solution incubated for 20 min. A PTN protein standard curve was always included. The resulting values were averaged and normalized to cell number and the length of exposure to the media.

### Immunoassays of cells and culture media

For immunofluorescence, cells were grown in culture dishes and secretion was blocked for 3 hr with the drug monensin to allow secreted proteins to accumulate in the Golgi apparatus [45]. The cells were then fixed in 1.5% formaldehyde in PBS for 15 min at room temperature (RT), followed by permeabilization and blocking in normal donkey serum staining buffer (2% normal donkey serum and 0.3% Triton-X100 in PBS with 0.02% sodium azide) for 30 min at RT. The cells were then incubated with the following primary antibodies: goat anti-hPTN (1∶50; R&D Systems), mouse anti-PDI to detect ER (1∶100; Abcam, Cambridge, MA), and rabbit anti-Gm130 to detect Golgi (1∶100; Abcam) in staining buffer for 1 hr, rinsed several times with staining buffer, and incubated with the following secondary antibodies (BD Pharmingen, San Jose, CA): donkey anti-goat Alexa 546, goat anti-mouse Alexa 350, and goat anti-rabbit Alexa 488 at 1∶200 dilution for 1 hr.

### Western blot analysis of CHOP expression

CHOP expression was assessed in T-PTN, PTN, and LacZ myoblasts. LacZ myoblasts treated with 5 µg/ml tunicamycin for 6 hr served as a positive control of ER stress. Cells were lysed with M-PER buffer (Thermo Scientific, Rockford, IL) plus 50 µl 1 M NaF and cleared lysates were electrophoresed on 10% NuPAGE gels (Invitrogen) and transferred to polyvinylidene difluoride membranes (Millipore). Blocking, antibody incubation, and washing were done in PBS with 0.1% Tween-20 (v/v) and 5% (w/v) non-fat dry milk. Primary antibodies were diluted: CHOP (1∶1000, Cell Signaling Technolgy, Beverly, MA), β-actin (1∶2000, Sigma) and incubated overnight. Then antibodies were detected by horseradish peroxidase-conjugated anti-mouse (1∶10000, Pierce) and visualized with a Pierce ECL Western Blotting Substrate Kit (Thermo Scientific).

### Ultrasound-guided injections into myocardium

The animal protocol was approved by the UCSF Institutional Animal Care and Use Committee, and was performed in accordance with the recommendations of the American Association Accreditation of Laboratory Animal Care. Briefly, SCID mice were anesthetized with 2% isoflurane and received closed-chest ultrasound-guided injection of PTN- or LacZ-expressing myoblasts (n = 5/group) into myocardium as previously described [37]. Hearts were injected with 1×10^6^ cells divided into two 5-µl injections into the anterior wall. All animals were judged to be optimally injected and none needed to be removed from the study due to poor injection into the LV cavity. Mice were euthanized at 14 or 28 days after implantation, and hearts were excised and cryosectioned.

### Tumorigenesis

To assess tumor growth in animals, male athymic nude mice (Ncr Nu, Taconic, Hudson, NY) were injected with 1×10^5^ PTN myoblasts or MDA-MB-231 breast cancer cells (generously provided by Andrei Goga, UCSF) in 10 ul PBS or Matrigel subcutaneously into the flanks and observed for 4 or 10 weeks. To evaluate contact inhibition of cell growth, 5×10^5^ myoblasts were plated in 6-well culture dishes (n = 4). Myoblast growth medium was changed every 48 hr. Cell number and percent viability by Trypan Blue staining were quantified using a hemocytometer.

### Ischemic hindlimb model and implantation of myoblasts into skeletal muscle

Unilateral hindlimb ischemia was induced in SCID mice (Jackson Laboratory, Bar Harbor, ME, 12–16 week old). Briefly, under sterile conditions, the superficial and deep femoral arteries were ligated and excised as described previously [46]. A sham procedure was performed on the contralateral leg. PTN myoblasts or LacZ myoblasts were injected once into the right tibialis anterior and once into the right lateral gastrocnemius in the normal mice or immediately after the ligation into the ischemic quadriceps as previously described [9], [47]. Each injection consisted of 5×10^5^ cells in 5 µl of PBS with 0.5% BSA. Animals were euthanized 2 weeks after surgery, and the injected muscles were harvested, embedded in OCT, and frozen in isopentane cooled in liquid nitrogen.

### Laser Doppler perfusion imaging of hindlimb blood flow

Mice were anesthetized using 1% isoflurane, after which their hindlimbs were shaved and then depilated using Surgiprep depilatory cream. The mice were put on a homeothermic heating pad and allowed to equilibrate to a rectal temperature of 37°C. Laser Doppler skin perfusion imaging was then performed over the legs and feet using a laser Doppler perfusion imager (Moor LDI; Moor instruments, Devon, UK). Measurements were recorded on the mid and distal portion of the adductor muscles of both legs before and after surgical ligation of arteries and on the day of euthanization. Perfusion was expressed as the ratio of ischemic/non-ischemic leg. The same area of interest (bilateral leg and foot) was scanned three times, and the results were averaged.

### Microsphere measurement of blood flow

Blood flow was measured using fluorescent microspheres as described previously [46]. Fourteen days after surgery, the chest was opened under isoflurane anesthesia through median thoracotomy, and 2×10^5^ 15 µm diameter blue-fluorescent microspheres (Molecular Probes, Eugene, OR) were continuously injected over 60 s into the beating left ventricle. The mid-thigh part of the leg muscle was excised, weighed, embedded in OCT-compound, and snap frozen. Lungs were excised as reference organs to confirm that microspheres were not injected into the right ventricle. Microspheres were individually counted by direct fluorescence microscopy on 100 µm cryosections from the entire sample [48], [49]. Microsphere counts from ischemic leg were normalized to counts from the contralateral, non-ischemic leg.

### Tissue histochemistry

Mice were sacrificed and muscle tissue was frozen in freezing isopentane, cryosectioned, and stained with X-gal (20 µm sections) or hematoxylin and eosin (H&E; 10 µm sections) as described previously [47]. For immunofluorescent staining of neighboring 10 µm sections, sections were fixed in 1.5% formaldehyde for 15 min, and then were blocked with normal donkey serum staining buffer for 30 min (2% normal donkey serum and 0.02% sodium azide and 0.3% Triton-X100 in PBS), then blocked with Biotin Blocking System (DAKO, Carpinteria, CA). Slides were incubated for 10 min at RT and then exposed to primary antibody for 1 hr, consisting of goat anti-human PTN (R&D Systems; 1∶50), mouse anti-smooth muscle actin monoclonal antibody (clone 1A4; ICN MP Biomedicals, Aurora, OH; 1∶400, and rabbit anti-β-gal (Eppendorf 5′; 1∶400), as well as biotin conjugated BS-1 isolectin B4 (Sigma-Aldrich, St Louis, MO; 1∶100). Negative controls lacking primary antibody were always performed. Sections were rinsed in staining buffer and then incubated for 1–1.5 hr at RT with donkey anti-goat Alexa 488, goat anti-mouse Alexa 488 antibody, or goat anti-rabbit Alexa 350 (1∶200), or streptavidin-conjugated Alexa 546 (1∶100; antibodies from BD Pharmingen). The slides were then rinsed and mounted, and viewed with a Nikon E800 fluorescence microscope using Openlab software (Improvision, Lexington, MA).

### Quantitation of blood vessels

Capillaries and arterioles were counted using a 20x objective within the implantation area. For capillaries, vessel area density and vessel length density indices were calculated as follows. A pattern consisting of repeating sine waves in a third color was imported into Openlab as a new layer, merged with each capillary photo, and the number of intersections of vessels with the pattern was counted. Vessel area density index was defined as the total number of intersections of capillaries and overlay pattern. Vessel length density was defined as the number of intersections of capillary centerlines and overlay pattern. To control for rips and gaps in the tissue, the number of sine wave peaks and troughs that occurred within tissue was counted for each field, and all vessel density indices were normalized to the number of tissue sine wave peaks/troughs to yield corrected indices for each field. Arteriole density was counted manually as the total number of arterioles per mm^2^ within the implantation area. All counts were blinded.

### Chemotaxis assay

Cell migration toward PTN was quantified by a transwell chemotaxis assay using a modified Boyden chamber according to our previously published conditions [19]. Briefly, chemotaxis was measured as follows: 16 chambers on two 24-well plates were pre-coated with 10% BSA, then rinsed. On each plate, 8 coated chambers were seeded with HUVECs at 1×10^4^ cells/cm^2^ and left to incubate overnight. 32 Transwell inserts with 8 um pores (Corning Transwell, Corning, NY) were coated in gelatin (0.05%) and vitronectin (50 ng/ul.) SMCs were resuspended in minimal media (EBM-2 without supplements +0.5% BSA) and 1×10^4^ were added to the upper chamber of each insert. Coated wells on each plate were treated in quadruplicate to assay migration towards minimal medium control, PTN (50 ng/ml,) HUVECs, and PTN + HUVECs. SMC-loaded inserts were placed above the treated chambers and plates, and were incubated separately in normoxic and hypoxic conditions (2% oxygen). After 6 h, the membranes were washed twice in PBS and fixed in 4% paraformaldehyde. After wiping cells off the upper side of each membrane with a cotton swab (Q-tip), the bottom sides were immersed briefly in the nuclear dye Hoechst 33342 (1∶4000 in PBS.) Membranes were detached from inserts via scalpel and mounted on glass slides. Migrated cells were counted on the lower side of the membrane by fluorescence microscopy. Chemotaxis was quantified as the net number of migrated cells (cell number/HPF with chemoattractant minus cell number/HPF without chemoattractant). Each experimental condition was performed in quadruplicate and the number of migrated cells was determined from 5 random 20×-fields (24.95 mm^2^) per membrane.

### Statistics

Data are presented as mean±SD. For the comparison within each group, Student's t-test was used and one way ANOVA was used for laser Doppler measurement. Group differences of migration assays were calculated with one way ANOVA and post hoc Tukey test, P<0.05 was considered significant.
